# Editorial for the Special Issue on the New Trends in Microwave/Millimeter Antennas/Filters: From Fundamental Research to Applications

**DOI:** 10.3390/mi14112019

**Published:** 2023-10-30

**Authors:** Ahmed A. Ibrahim, Syed Muzahir Abbas

**Affiliations:** 1 Electronics and Communications Engineering Department, Minia University, El-Minia 61519, Egypt; 2 School of Engineering, Faculty of Science and Engineering, Macquarie University, Sydney, NSW 2109, Australia

The rapid growth of wireless communication systems has led to high demand for the design of microwave/millimeter components with multiband characteristics, high performance, and ease of integration with other devices [1,2,3]. Recently, 5G wireless communication networks have started to stimulate the development of beam-steering techniques [4,5,6]. In comparison with previous technologies, including 4G wireless applications, 5G is shifting to higher frequencies to offer wider bandwidths and provide a higher capacity [7,8,9]. The use of millimeter wave and sub-6 GHz bands has been proposed to benefit services supporting networks of small/large cells facilitating high-capacity hotspot zones while increasing area efficiency [10,11,12]. 

In this Special Issue, we target the latest technology and developments in microwave/millimeter system components and aim to overcome technical challenges by bringing together academics and industrial researchers to identify and discuss novel results within this continuously evolving field. The goal of this Special Issue is to stimulate the community by addressing the key issues on the topic in the hope that antennas and filters will make a greater impact in our society. The 19 published papers in this volume have presented cutting-edge research findings. In total, 17 papers have discussed antennas, MIMO antennas, and RFID, and two of them discussed filters. The submitted papers are from authors from different countries, including Australia, China, Egypt, India, Iraq, Ireland, Italy, Jordan, Malaysia, Morrocco, Pakistan, Palestine, Saudi Arabia, Spain, Turkey, and the United Kingdom. Novel techniques have been reported in this issue for the development of filters and antennas. 

Multiple-input multiple-output (MIMO) antennas are of great interest due to their attractive features. Five published papers have discussed the design of MIMO antennas for 5G, ultra-wideband (UWB), and other wireless applications. Ultra-wideband (UWB) technology is widely used in many communication scenarios. However, narrowband systems can easily interfere with a UWB system, which generates multipath fading. In order to solve these interferences and meet the design requirements of high isolation of multiple-input multiple-output (MIMO) antennas, two MIMO antennas with double-notch structures are designed (contribution 1). Firstly, two U-shaped slots are etched on the radiating patch and feeder to achieve notch characteristics in WiMAX and ITU bands. Using this antenna element, a two-element antenna is put symmetrically in parallel, and two rectangular branches are loaded to improve the isolation. The size is 0.57λ × 0.32λ × 0.013λ (at 2.5 GHz). Then, a four-element antenna is designed to meet the requirements for another application; here, each element is placed orthogonally to each other, and the isolation is improved by loading a cross-shaped branch in the middle of these elements. The size is 0.57λ × 0.57λ × 0.013λ. Both antenna samples are tested to verify the design. The measurement results show that the working bandwidths are 2.45–14.88 GHz and 2.14–14.95 GHz, the isolations are greater than 17 and 20 dB, and the peak gains are 5.7 and 5.9 dBi for the two- and four-element MIMO antennas, respectively. Compared to the references, the designed antennas have a wider bandwidth and a higher gain and radiation efficiency. They are well-suited for diverse wireless applications. 

In another work, a 4 × 4 miniaturized UWB-MIMO antenna using FR-4 material with reduced isolation was designed and analyzed using characteristic mode analysis (contribution 2). To minimize the antenna’s physical size and to improve the isolation, an arrangement of four symmetrical radiating elements was positioned orthogonally. The antenna dimension was 40 mm × 40 mm (0.42λ_0_ × 0.42λ_0_) (λ_0_ was the wavelength at the first lower frequency). A square-shaped defected ground framework was placed on the ground to improve the isolation. Etching square-shaped slots on the ground plane achieved the return losses S11 < −10 dB and a 26 dB isolation in the entire operating band of 3.2–12.44 GHz (UWB (3.1–10.6 GHz) and the X-band (8–12 GHz) spectrum and achieved good isolation bandwidth of 118.15%. The MIMO performance was evaluated in terms of diversity gain (DG), envelope correlation coefficient (ECC), Channel Capacity Loss (CCL), and mean effective gain (MEG).

You et al. presented a 12-port MIMO antenna system for 5G/WLAN applications (contribution 3). The proposed antenna system consisted of two types of antenna modules: an L-shaped antenna module covering the C-band (3.4–3.6 GHz) for 5G mobile applications and a folded monopole module for the 5G/WLAN mobile application band (4.5–5.9 GHz). Each two antennas formed a pair, six pairs in total, forming a 12 × 12 MIMO antenna array, and the elements between the antenna pairs could achieve an isolation of 11 dB or more without additional decoupling structures. The experimental results showed that the antenna could cover the 3.3–3.6 GHz and 4.5–5.9 GHz bands with an overall efficiency greater than 75% and an envelope correlation coefficient of less than 0.04. Finally, the one-hand holding mode and two-hand holding mode were discussed to demonstrate their stability in practical applications, and the results showed that they still exhibited good radiation and MIMO performances when operating in both modes. 

Another design of multiband MIMO antennas along with high-isolation characteristics using FR-4 material has been proposed (contribution 4). It operates at the 3.50 GHz, 5.50 GHz, and 6.50 GHz frequencies for 5G cellular, 5G WiFi, and WiFi-6, respectively. The two-element MIMO multiband antenna was miniaturized to 16 × 28 × 1.6 mm^3^, making it desirable for devices operating in 5G bands. High isolation (>15 dB) was attained with thorough testing without employing a decoupling scheme in the design. Laboratory measurements resulted in a peak gain of 3.49 dBi and an efficiency of around 80% in the entire operating band. The evaluation of the presented MIMO multiband antenna was carried out in terms of ECC, DG, total active reflection coefficient (TARC), and CCL. The measured ECC was less than 0.04, and the DG was well above 9.50. The observed TARC was also lower than −10 dB, and the CCL was below 0.4 bits/s/Hz in the entire operating band.

Al-Khaylani et al. presented a novel design of a reconfigurable MIMO antenna array of a 3D geometry-based solar cell integration that operated at sub-6 GHz for self-powered applications in a 5G modern wireless communication network (contribution 5). The proposed antenna array provided three main frequency bands around 3.6 GHz, 3.9 GHz, and 4.9 GHz, with excellent matching impedance of S11 ≤ −10 dB. The proposed MIMO array was constructed from four antenna elements arranged on a cubical structure to provide a low mutual coupling, below −20 dB, over all frequency bands of interest. Each antenna element was excited with a coplanar waveguide (CPW). The proposed radiation patterns were controlled with two optical switches of Light-Dependent Resistors (LDRs). The proposed antenna array was fabricated and tested experimentally in terms of S-parameters, gain, and radiation patterns. The maximum gains were found to be 3.6 dBi, 6.9 dBi, and 3.5 dBi at 3.6 GHz, 3.9 GHz, and 4.9 GHz, respectively. It was realized that the proposed array realized significant beam forming by splitting the antenna beam and changing the main lobe direction at 3.9 GHz after changing LDR switching statuses. Such an antenna array was found to be very applicable for femtocell wireless communication networks in 5G systems.

In this Special Issue, three of the published papers are related to reconfigurable antennas and their applications for wireless communications. The first paper presents a reconfigurable wideband monopole antenna for cognitive radio and wireless applications (contribution 6). The reconfigurability was achieved by four varactor diodes embedded in the band pass filter (BPF) structure, which was integrated with the suggested antenna through its feed line. The simulated impedance characteristics coped with the measured characteristics after fabricating the suggested model with/without the reconfigurable BPF. Furthermore, the model achieved the desired radiation characteristics in terms of radiation pattern with acceptable gain values at the selected frequencies within the achieved frequency range (1.3–3 GHz). 

The second paper presents a novel antenna structure constructed from cascading multi-stage metamaterial (MTM) unit cells-based printed monopole antenna for 5G mobile communication networks (contribution 7). The proposed antenna is constructed from a printed conductive trace that fetches four MTM unit cells through four T-Resonators (TR) structures. Such a combination is introduced to enhance the antenna gain-bandwidth products at sub-6GHz bands after exiting the antenna with a coplanar waveguide (CPW) feed. The antenna circuitry is fabricated by etching a copper layer that is mounted on a Taconic RF-43 substrate and has an effective area of 51 × 24 mm^2^. The proposed antenna provides an acceptable matching impedance with S11 ≤ −10 dB at 3.7 GHz, 4.6 GHz, 5.2 GHz, and 5.9 GHz. The antenna radiation patterns are evaluated at the frequency bands of interest with a gain average of 9.1–11.6 dBi. Moreover, four optical switches based on LDR resistors are applied to control the antenna gain at 5.85 GHz, which is found to vary from 2 dBi to 11.6 dBi after varying the value of the LDR resistance from 700 Ω to 0 Ω, in a descending manner. It has been found that the proposed antenna provides an acceptable bit error rate (BER) with varying the antenna gain in a very acceptable manner in comparison to the ideal performance. Finally, the proposed antenna is fabricated to be tested experimentally in free space and close to the human body for portable applications. Similarly, the third design introduced a reconfigurable MIMO antenna array of a 3D geometry-based solar cell integration that operates at sub-6 GHz for self-power applications in a 5G modern wireless communication network (contribution 5). The antenna radiation patterns are controlled with two optical switches of Light-Dependent Resistors (LDRs).

Two published papers in this Special Issue have discussed dielectric resonator antennas (DRAs) to achieve circular polarization behavior. The first design discussed a wide dual-band circularly polarized (CP) operation for deployment in indoor radio links and INSAT applications (contribution 8). The metasurface and DRs are hosted above a grounded substrate, which is fed through a single coaxial feed placed at a specific angle, employing a modified upper probe of the coaxial fee to enhance the antenna performance. The proposed hybrid technique utilizes the combined benefits of the feed angle and a well-matched metasurface, resulting in performance improvements. Notably, a measured impedance bandwidth of 88.1% for |S11|is achieved within the frequency range of 4.0 GHz to 10.3 GHz. Furthermore, the antenna design exhibits two overlapping, measured at 3 dB axial ratio (AR) bandwidths: 23.62% from 4.25 GHz to 5.4 GHz and 5.12% from 7.6 GHz to 8 GHz. The peak gain of the antenna is measured at 8.4 dBic. The second design presented a novel approach to design a circularly polarized (CP) hemispherical dielectric resonator antenna with a wide axial ratio (AR) bandwidth by incorporating an additional dielectric substrate between the antenna and the ground plane for 5G communication (contribution 9).

In addition to the above-mentioned research work, in this Special Issue, nine novel antennas and filter designs have been presented for various wireless and 5G communication technologies. Zhai et al. proposed a quasi-Yagi antenna with resistor-loaded arms to obtain a filtering response with four radiation nulls (contribution 10). The embedded resistor-loaded arms achieved two additional radiation nulls caused by reverse currents and absorbed the unwanted out-of-band resonant points brought by themselves. The director close to the driver provided a resonant point and a radiation null caused by opposite currents between the driver and the director. Compared with other filtering quasi-Yagi antennas, the proposed design could achieve a filtering response with a compact size along the end-fire direction. A balun-integrated prototype covering the 5G band N78 (3.3–3.8 GHz) showed a 10 dB impedance-matching bandwidth of 22.9% (3.21–4.04 GHz), four radiation nulls, and a peak gain of 4.73 dBi. Another design introduced a high-gain UWB Vivaldi antenna loaded with artificial electromagnetic material, suitable for ground-penetrating radar (GPR) systems (contribution 11). The artificial electromagnetic units affected the antenna radiation waves by changing the refractive index to enhance the antenna’s directivity. Moreover, four Vivaldi units were arranged into a horn-shaped array, and each of the two units were orthogonally fed to realize dual polarization.

A compact-size UHF RFID tag antenna using slot apertures and capacitive gaps with large reads was ranged for outdoor localization applications (contribution 12). The effects of capacitive slots and gaps on the impedance matching between conventional industrial chips and a designed RFID antenna were investigated. Alternatively, a multi-frequency antenna loaded with a ring structure has been presented (contribution 13). The patch consists of resonator structures, and the ground plate consists of a bottom metal strip and three ring-shaped metals with regular cuts to form a defective ground structure to be operated for 5G NR (FR1, 0.45–3 GHz), 4GLTE (1.6265–1.6605 GHz), Personal Communication System (1.85–1.99 GHz), Universal Mobile Telecommunications System (1.92–2.176 GHz), WiMAX (2.5–2.69 GHz), and other communications frequency bands. Similarly, another design introduced a multiband planar inverted L-C implantable antenna with SAR values within the safety limits with maximum allowable input power (contribution 14). The proposed antenna operates at low power levels and supports an energy-efficient solution. Additionally, the SAR values are within the safety limits, with a maximum allowable input power (8.43 mW (1 g) and 47.5 mW (10 g) at 402.5 MHz; 12.85 mW (1 g) and 47.8 mW (10 g) at 2.45 GHz; and 11 mW (1 g) and 50.5 mW (10 g) at 2.95 GHz). A wideband graded effective refractive index (GRIN) dielectric lens antenna for 5G mm-wave band applications has been presented (contribution 15). The inhomogeneous holes in the dielectric plate are perforated to provide GRIN in the proposed lens.

A UWB monopole antenna with an octagonal patch was fed with a 50 Ω line and with a triple band notch feature (contribution 16). In this design, an L-shaped stub, an inverted C-shaped slot, and a pair of U-shaped resonating structures are introduced into the design, which allows the antenna to generate three band notches at 3.22–3.83 GHz, 4.49–5.05 GHz, and 7.49–8.02 GHz, corresponding to the WiMAX band, Indian national satellite (INSAT) band, and X-band satellite frequencies, respectively. Dayo et al. presented a miniaturized high-performance flower-shaped radiator (FSR) (contribution 17). The antenna can be used for modern smartphones’ connectivity with the sub-6 GHz frequency spectrum of modern 5G mobile communication applications. Furthermore, an antenna pair for mobile terminals using a type of coupling feed planar antenna and a wide band coupling suppression has been proposed (contribution 18). The process of band expansion comes from a dual-band antenna pair and is based on characteristic modes theory (CMA). The multiple defective ground structure (DGS) is introduced for isolation enhancement. Another work has introduced a flexible method for designing a bandpass filter (BPF) using pixel structure and genetic algorithm (GA) optimization (contribution 19). The pixel structure is made up of a grid of metallic microstrip stubs, and the GA is utilized to determine the connections between these stubs. Furthermore, these designs are designed, fabricated, and measured with good trends between the outcomes, which support their ideas to be utilized in different wireless applications.

We hope that this Special Issue on antennas and filters will offer readers a good overview of the current state-of-the-art developments in these fast-growing areas of research, as well as an introduction to some of the newest techniques developed in this field.
